# Spigelian hernia in the right upper abdominal wall: a case report

**DOI:** 10.1186/s12893-018-0449-5

**Published:** 2018-11-27

**Authors:** Zhou Ye, Mo-Jin Wang, Li-Fen Bai, Han-Xiang Zhuang, Wen Zhuang

**Affiliations:** 1 Department of Digestive Diseases, The Third Affiliated Hospital of Chongqing Medical University (Gener Hospital), Chongqing, China; 2 Department of Gastrointestinal Surgery, West China Hospital, Sichuan University, 37 Guo Xue Xiang, Chengdu, 610041 Sichuan Province China

**Keywords:** Spigelian hernia, Spontaneous, Strangulation, Surgery

## Abstract

**Background:**

Spigelian hernia (SH) is rare and constitutes less than 2% of all hernias. It is reported that more than 90% of SHs lie in the “Spigelian belt”, but SH in the upper abdominal wall is extremely uncommon. Here, we report a case of SH in the right upper quadrant of abdomen.

**Case presentation:**

A 38-year-old female was admitted to hospital with complaints of abdominal pain and right upper quadrant mass for 10 days. Contrast-enhanced computed tomography (CECT) of abdomen revealed the dilated small intestine between the swelling ventral muscles in the right upper abdominal wall which suggested a ventral hernia. The surgeons considered it was a spontaneous hernia because there was no history of surgery or trauma in the upper abdomen. About two hours later, the patient underwent emergency surgery. According to laparotomy, a diagnosis of SH with ileum herniation in the right upper abdominal wall was confirmed. The necrotic ileum segment was resected. Meanwhile the abdominal wall defect was repaired by suturing the internal oblique and transverse muscles to the rectus sheath. The patient had a favorable outcome for 1 year without recurrence.

**Conclusion:**

A mass and pain in the upper abdominal wall may suggest an atypical SH. SH occurring in the upper abdominal wall is a rare condition with possibility of dire outcome if not managed early.

**Electronic supplementary material:**

The online version of this article (10.1186/s12893-018-0449-5) contains supplementary material, which is available to authorized users.

## Background

**Fig. 1:**
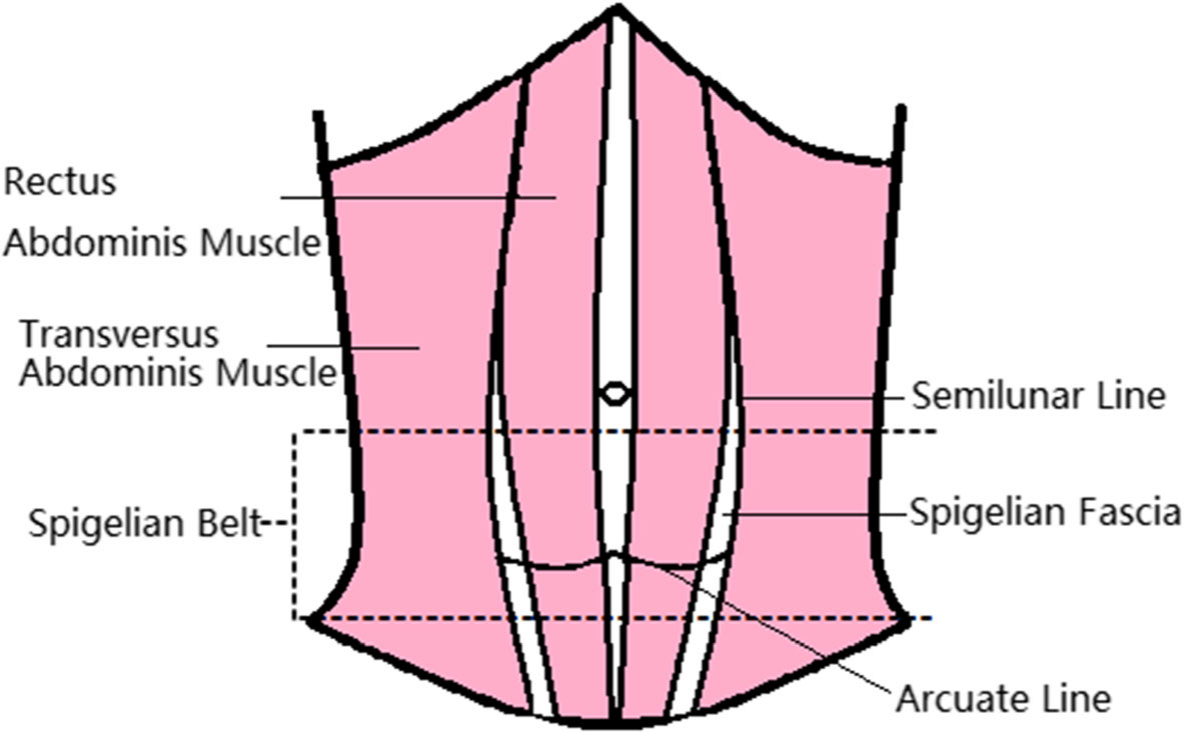
The location of Spigelian fascia and “Spigelian belt” (The image in Fig. 1 was drawn by Zhou Ye)

Spigelian hernia (SH) is named after Adriaan van Spieghel, who depicted the semilunar line in 1645 [1]. The semilunar line is defined as the transition of the transversus abdominis muscle to its aponeurotic tendon [2]. Spigelian fascia is located between the semilunar line and the lateral edge of the rectus abdominis muscle. SH is a spontaneous abdominal hernia caused by a defect in the Spigelian fascia [3]. The hernia is rare and constitutes less than 2% of all hernias [4]. SH occurs anywhere on the Spigelian fascia, but it is reported that more than 90% of these hernias are located in the “Spigelian belt”, which is a transverse 6-cm-wide zone in the lower abdominal wall (Fig. 1) [5]. However, SH occurring in the upper abdominal wall is extremely rare. We present a case of SH in the right upper quadrant.

## Case presentation

**Fig. 2:**
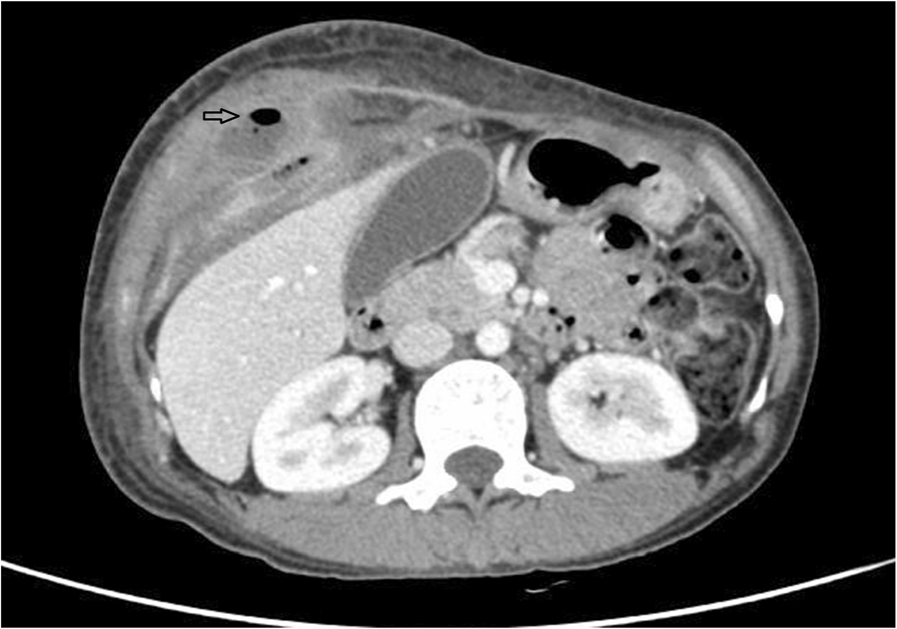
CECT investigation revealed the presence of air between ventral muscles in the right upper abdominal wall

**Fig. 3:**
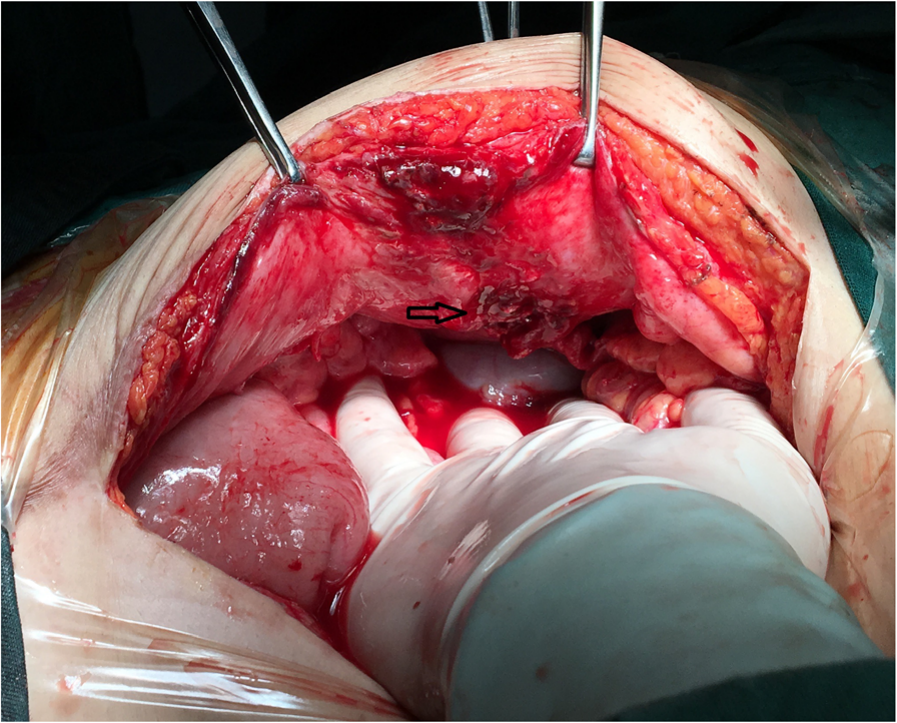
The hernia sac (arrow) was located between the semilunar line and the edge of the rectus abdominis muscles

A 38-year-old female patient presented with abdominal pain and right upper quadrant mass for 10 days. She had a history of pregnancy and caesarean section in the lower abdominal region 10 years before, but there was no history of trauma and upper abdominal surgery. Clinical examination showed a huge mass about 15 × 10 cm over right upper abdomen. There was moderate tenderness in the area of the mass, and the abdominal skin was intact and smooth except the lower abdominal surgical scar. Abdominal wall ultrasonography showed mixed echo-mass under the muscle layer of right upper abdominal wall. Contrast-enhanced computed tomography (CECT) of abdomen was performed. The images revealed the dilated small intestine between the swelling ventral muscles in the right upper abdominal wall which suggested a ventral hernia in the right upper abdominal wall (Fig. 2). There was no history of right upper abdominal surgery or trauma, therefore the surgeons considered it was a spontaneous hernia. The patient had much more severe abdominal pain and began to present hematochezia during her hospitalization. Two hours later, she underwent emergency surgery. The intraoperative finding was an atypical ventral hernia, and the ileum loop was incarcerated with necrosis. The hernia sac was located between the semilunar line and the edge of the rectus abdominis muscles in the right upper abdominal wall (Fig. 3), which confirmed it was a Spigelian hernia. The defect size was 2.0 × 1.5 cm. The necrotic ileum segment was resected and end-to-end anastomosis was performed. Considering the small defect and the necrotic ileum, a simple herniorrhaphy was made by suturing the internal oblique and transverse muscles to the rectus sheath. The postoperative course was uneventful, the patient recovered well and was discharged after 1 week of hospitalization. The patient had a favorable outcome for 1 year without recurrence.

## Discussion

Spigelian hernia (SH), also known as spontaneous lateral ventral hernia, usually occurs when abdominal pressure increases. SHs in most cases have explicit predisposed causes, such as chronic coughing, obesity, peritoneal dialysis [6–12]. The site of the hernia is a significant indicator to identify SH. The Spigelian fascia is wider beneath the umbilical region, so it is weaker than that in the upper abdomen. However, superior to umbilical region, the transverses abdominis muscle and internal oblique muscle often extend into the posterior rectus sheath which makes the Spigelian fascia stronger [3]. Anatomically, SH occurring in the upper abdomen is uncommon. Furthermore, in the upper abdominal wall, the Spigelian fascia is posterior to the rectus muscle, making it difficult even for the experienced surgeon to locate a fascial defect during the physical examination.

Ultrasound is recommended as first line imaging investigation, and CT scanning should be added in challenging cases [13–15]. Other studies show that the CT scanning is better than ultrasound, because ultrasound is dependent by the operator [11, 16]. Still, it is reported that only 50% of cases are correctly diagnosed preoperatively [17].

SH is dangerous and the risk of incarceration is higher than other hernias because the defect can be small. It is reported that the risk of incarceration is up to 21%, and thus patients should be offered prompt surgical repair [18]. Surgical procedures are generally classified as open and laparoscopic procedures. The laparoscopic approach should be applied in uncomplicated cases [19, 20]. If the defect is extensive(usually more than 5 cm) and the abdominal wall is obviously destructive, open surgery should be performed [3]. Repairing the defect of SH contains fascial closure or fascial suturing reinforced with synthetic mesh in the cases of large defects [21, 22]. Small hernia defects could be repaired by laparoscopic herniorrhaphy alone [23].

In conclusion, a mass and pain in the upper abdominal wall may suggest an atypical SH. In addition, the risk of strangulation of SH is higher than other hernias. SH occurring in the upper abdominal wall is a rare condition with possibility of dire outcome if not managed early.

## Additional files

Additional file 1: CT 1. CECT investigation revealed the ventral hernia (transverse section 1). (TIF 73 kb)

Additional file 2: CT 2. CECT investigation revealed the ventral hernia (transverse section 2). (TIF 73 kb)

## Abbreviations

CECT: Contrast-enhanced computed tomography
CT: Computed tomography
SH: Spigelian hernia
