# Supplementary figures and images for: Spigelian hernia in the right upper abdominal wall: a case report
Source: BMC Surg. 2018 Nov 27;18:109. doi: 10.1186/s12893-018-0449-5 (PMC6260715; doi:10.1186/s12893-018-0449-5)

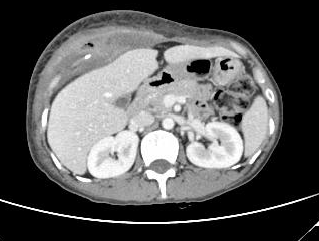

Supplement: Supplementary file 1 — CT 1. CECT investigation revealed the ventral hernia (transverse section 1). (TIF 73 kb) [file 12893_2018_449_MOESM1_ESM.tif]

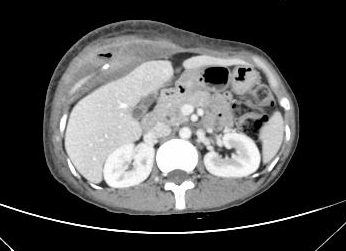

Supplement: Supplementary file 2 — CT 2. CECT investigation revealed the ventral hernia (transverse section 2). (TIF 73 kb) [file 12893_2018_449_MOESM2_ESM.tif]
